# Identification, synthesis and regulatory function of the *N*-acylated homoserine lactone signals produced by *Pseudomonas chlororaphis* HT66

**DOI:** 10.1186/s12934-017-0854-y

**Published:** 2018-01-22

**Authors:** Huasong Peng, Yi Ouyang, Muhammad Bilal, Wei Wang, Hongbo Hu, Xuehong Zhang

**Affiliations:** State Key Laboratory of Microbial Metabolism, School of Life Sciences and Biotechnology, Shanghai Jiao Tong University, 800 Dongchuan Road, Shanghai, 200240 People’s Republic of China

**Keywords:** *Pseudomonas chlororaphis*, Phenazine-1-carboxamide, Quorum sensing, *N*-Acylated homoserine lactones, *phzI*, Biofilm formation

## Abstract

**Background:**

*Pseudomonas chlororaphis* HT66 isolated from the rice rhizosphere is an important plant growth-promoting rhizobacteria that produce phenazine-1-carboxamide (PCN) in high yield. Phenazine production is regulated by a quorum sensing (QS) system that involves the *N*-acylated homoserine lactones (AHLs)—a prevalent type of QS molecule.

**Results:**

Three QS signals were detected by thin layer chromatography (TLC) and high-performance liquid chromatography–mass spectrometry (HPLC–MS/MS), which identified to be *N*-(3-hydroxy hexanoyl)-l-homoserine lactone (3-OH-C6-HSL), *N*-(3-hydroxy octanoyl)-l-homoserine lactone (3-OH-C8-HSL) and *N*-(3-hydroxy decanoyl)-l-homoserine lactone (3-OH-C10-HSL). The signal types and methods of synthesis were different from that in other phenazine-producing *Pseudomonas* strains. By non-scar deletion and heterologous expression techniques, the biosynthesis of the AHL-signals was confirmed to be only catalyzed by *Phz*I, while other AHLs synthases i.e., *Csa*I *and Hdt*S were not involved in strain HT66. In comparison to wild-type HT66, PCN production was 2.3-folds improved by over-expression of *phz*I, however, *phz*I or *phz*R mutant did not produce PCN. The cell growth of HT66∆phzI mutant was significantly decreased, and the biofilm formation in *phz*I or *phz*R inactivated strains of HT66 decreased to various extents.

**Conclusion:**

In conclusion, the results demonstrate that *Phz*I–*Phz*R system plays a critical role in numerous biological processes including phenazine production.

**Electronic supplementary material:**

The online version of this article (10.1186/s12934-017-0854-y) contains supplementary material, which is available to authorized users.

## Background

Quorum sensing (QS) is a well-studied form of communication process used by a large variety of bacteria to regulate diverse cellular functions such as antibiotic production, biofilm development, gene expression, surface attachment and virulence in a cell-population density-dependent manner [1, 2]. The Gram-negative bacteria use *N*-acylated homoserine lactones (AHLs) to sense cell density, which is composed of a homoserine lactone ring (HSL) with varying acyl chain [3]. These diffusible small signaling molecules are synthesized by a member of the LuxI protein family. Bacteria can monitor cell-population density by measuring the concentration of small secreted signaling molecules, so-called AHLs. When AHL concentration reaches a certain threshold value, the cells can switch on the expression of a set of genes responsible for the production of bioluminescence [4], antibiotics [5], plasmid transfer [6], and symbiosis [7] etc. Therefore, the AHL-mediated QS play an essential role in numerous biological processes.

Phenazines are a class of pigmented heterocyclic metabolites, which are produced by the genera *Pseudomonas*, *Burkholderia*, and *Streptomyces* [8]. Phenazine and its derivatives, such as a phenazine-1-carboxylic acid (PCA), phenazine-1-carboxamide (PCN), and 2-hydroxy-phenazine (2-OH-PHZ), exhibit potent antifungal activities against a wide range of eukaryotic microbes and therefore could be employed as a fungicide in agriculture production [9]. For example, PCA greatly reduces the risk of a severe wheat root disease caused by *Gaeumannomyces graminis* var. *tritici* [10, 11]. PCN possesses notable antifungal activity against *Fusarium oxysporum* f. sp. *radices*-*lycopersici* [12, 13]. Intriguingly, almost all of the phenazine compounds secreted by bacteria display antimicrobial, anti-tumor, antimalarial, and antiparasitic effects compared with some chemically synthesized phenazine derivatives [14–19]. In addition, natural phenazine products show great promise for use as electron acceptors and donors, components of microbial fuel cells (MFC), and environmental sensors and biosensors [20–23]. Most phenazine-producing microorganisms have been isolated from diverse terrestrial, freshwater, and marine environments [24, 25]. Fluorescent pseudomonads, which are members of the gamma subclass of the proteobacteria, are the best-studied phenazine producers, with strains of *Pseudomonas fluorescens*, *P. chlororaphis*, and *P. aeruginosa* known to produce these compounds [26]. PhzI, a LuxI homolog, is an AHL synthase mainly responsible for the synthesis of AHL signals in phenazine-producing *Pseudomonas*. Subsequently, the AHL-receptor protein, PhzR, binds to AHL-signals and activates the expression of downstream phenazine biosynthetic gene cluster [27].

In this study, an isolated microbe from rice rhizosphere namely *Pseudomonas chlororaphis* HT66 has been selected. Based on whole genome sequencing, three possible AHL synthesis genes *phzI*, *csaI* and *hdtS* were investigated. Among them, the *phzI* gene is located upstream of the *phz* cluster like other *Pseudomonas*. However, the types and biosynthesis of AHL signals are unclear and the regulation of phenazine biosynthesis needs to be studied. The AHLs were identified by Thin Layer Chromatography (TLC) and high-performance liquid chromatography-mass spectrometry (HPLC–MS/MS) analyses. Additionally, we elucidated the relationship between phenazine production, biofilm formation and AHLs biosynthesis, including which gene is responsible for the AHLs synthesis. The biocontrol activity of *P. chlororaphis* HT66 and its derivatives were also evaluated.

## Methods

### Bacterial strains and growth conditions

Selected bacterial strains and plasmids used in this study are summarized in Table 1. Bacterial strains were grown in Luria–Bertani (LB) medium at 37 °C (for *Escherichia coli*) or King’s B (KB) medium at 28 °C (for *P. chlororaphis* HT66). The bioreporter strains, *Agrobacterium tumefaciens* NTL4 (pZLR4) [28] and *Chromobacterium violaceum* CV026 [29], were grown at 28 °C in AB minimal medium (ABM) [30] and LB medium, respectively. When required, antibiotics were used at the following concentrations: ampicillin (100 μg/ml), kanamycin (50 μg/ml) or tetracycline (20 μg/ml) for *E. coli* and ampicillin (100 μg/ml), kanamycin (50 μg/ml) and tetracycline (200 μg/ml) for *P. chlororaphis*; whereas the gentamicin (30 μg/ml) and kanamycin (20 μg/ml) were used for NTL4 (pZLR4) and CV026, respectively.

**Table 1:** Bacterial strains, and plasmids used in this study

Strain, plasmid	Characteristics	Reference or source
*Escherichia coli* strains		
*E.coli* DH5α	*supE44∆lacU169(ɸ80lacZ∆M15)recAhsdR17 recA1 endA1 gyrA96 thi-1 relA-1*	Hanahan [45]
*E. coli* S17	res^-^ pro mod^+^ integrated copy of RP4, mob^+^, used for incorporating constructs in *P. chlororaphis*	Hoffmann et al. [46]
*A. tumefaciens* NTL4 (pZLR4)	Biosensor strain for AHLs	Cha et al. [28]
*C. violaceum* CV026	Biosensor strain for AHLs	McClean et al. [29]
*Pythium ultimum*	The pathogen causes ripe fruit rot of tomato	Pearson et al. [33]
*Pseudomonas chlororaphis*		
HT66	PCN, wild-type, Amp^r^Sp^r^	This study
∆*phz*I	HT66 derivative, *phz*I deleted	This study
∆*phz*R	HT66 derivative, *phz*R deleted	This study
∆*csa*I	HT66 derivative, *csa*I deleted	This study
∆*hdt*S	HT66 derivative, *hdt*S deleted	This study
Plasmids		
pK18mobsacB	Broad-host-range gene replacement vector; sacB, Km^r^	Schafer et al. [47]
pME6032	IPTG-inducible expression vector, Tc^r^	Heeb et al. [48]
pME-*phz*I	*phz*I from HT66 in pME6032,Tc^r^	This study
pME-*csa*I	*csa*I from HT66 in pME6032,Tc^r^	This study
pME-*hdt*S	*hdt*S from HT66 in pME6032,Tc^r^	This study

^r^ resistance

### DNA manipulation and mutant construction

The non-scar gene deletion was carried out as previously described [31]. First, two pairs of primers designed to clone 100–600 bp fragments located upstream or downstream of the target gene. These two 100–600 bp fragments were amplified by polymerase chain reaction (PCR) from the genomic DNA of HT66. Next, an overlap PCR was used to combine the two fragments, which created a new sequence excluding the target gene. After digesting the overlap PCR product by restriction enzymes *Eco*RI and *Hin*dIII, the new sequence was ligated into pK18mobsacB by T4 DNA ligase. The resulting plasmid was first transferred into *E. coli* S17 and then mobilized into HT66 by conjugation. Afterwards, the colony carrying pK18mobsacB was inoculated into 15% sucrose counter-selection plate, only the marked mutant population and spontaneous Suc^R^ colonies grew. Finally, the colonies were tested and verified by PCR analysis and sequencing, to make sure a double crossover had occurred and the target part had been replaced by the new sequence. All the other gene deletions were carried out following the same strategy.

Taking the over-expression and complementation of *phzI* as an example, we used primers to amplify the 591-bp *phzI* gene from HT66. The resulting PCR-amplified fragment was digested with restriction enzymes *EcoR*I and *Xho*I and then cloned into vector pME6032, yielding pME-*phzI*. Through electroporation, plasmid pME-*phzI* was separately transformed into wild-type HT66 and ∆*phzI* for over-expression and trans-complementation. In a similar way, pME-*csaI* and pME-*hdtS* were also constructed.

### Extraction of AHLs from culture supernatants

Strain HT66 was grown in KB medium (enrichment media for *Pseudomonas*) at 28 °C for 24 h with shaking at 180 rpm. For the expression of *PhzI*, *CsaI* and *HdtS* in *E. coli*, strains were grown in LB medium to an OD_600_ of 0.1 and induced with Isopropyl *β*-d-1-Thiogalactopyranoside (IPTG) (final concentration, 1 mM) for 4–8 h. After growth, the bacterial cells were eliminated by centrifugation (8000×*g* for 10 min) and culture supernatants were extracted twice with equal volumes of ethyl acetate. The organic phase was pooled, dried over anhydrous magnesium sulfate. The ethyl acetate was removed by reduced pressure distillation at 30 °C and the residue contained the AHL extracts. The residue was stored at − 20 °C or dissolved with an appropriate volume of HPLC-grade acetonitrile for bioassay or HPLC–MS/MS analysis.

### TLC separation and visualization of AHLs

The AHL plate assay using *A. tumefaciens* NTL4 was performed as reported earlier [30]. Briefly, AHL extracts (1–5 μl), were applied to C18 reversed-phase (RP)-TLC plates (Merck, Germany) and the chromatograms were developed with methanol/water (60:40, *v/v*). The plates were removed from the chromatography tank when the solvent front reaches the top of the TLC plate. The air-dried plates were overlaid with ABM medium soft gel (0.8% agar), followed by the addition of X-Gal (5-Bromo-4-chloro-3-indolyl-*β*-d-galactopyranoside; 60 μg/ml) and overnight culture of *A. tumefaciens* NTL4. After the agar solidification, the chromatography plates were incubated at 28 °C for 12–18 h and observed the blue spots indicating the location of AHLs. TLC analysis was repeated at least three times.

### Analysis of AHLs by HPLC–MS/MS

AHL extracts from 100 ml cultures were partially purified by preparative TLC. For this, preparative TLC plate was cut into small strips and the compounds were carefully removed by scraping off the silica gel at the appropriate R_f_ and extracted three times with ethyl acetate. The dissolved compounds were centrifuged (at 12,000*g* for 10 min), and clear supernatant thus obtained was filtrated by syringe filters (0.22 µm). The supernatant was concentrated and the residue was dried and re-dissolved in acetonitrile for HPLC–MS/MS analysis at Instrumental Analysis Center of Shanghai Jiao Tong University, Shanghai China. A reverse phase column (5 μm; 4.6 × 250 mm, Shimadzu, Japan) with a detection wavelength of 254 nm was used. The AHL separation was performed with water containing 0.1% formic acid (analytical grade) and acetonitrile (AcN) (HPLC-grade). A flow rate of 0.5 mL/min was used, with increasing concentrations of 5 to 95% AcN in 15 min. Then, the flow was held for 3 min, and equilibration was performed for 7 min. All the mass spectra were recorded in the positive-ion mode. MS parameters were a spray voltage of 5 kV, a capillary temperature of 230 °C, and a sheath gas rate of 12 units N2.

### Determination of the PCN production

HT66 and its mutants were grown overnight and inoculated into the fresh KB medium (1% inoculum ratio) followed by incubation for 24 h at an agitation speed of 180 rpm. Fermentation broth (300 μl) was acidified to pH 2.0 with 6 M HCl, and then 2.7 ml of ethyl acetate was added. The sample was vigorously agitated and centrifuged at 13,000*g* for 5 min. A 300-μl portion of the upper layer was collected and evaporated to complete dryness in a rotary evaporator. The resulting residue was dissolved in 1 ml acetonitrile and PCN concentration was determined by HPLC (Agilent Technologies 1200 series, Santa Clara, USA) with mobile phase acetonitrile (component A) and 5 mM ammonium acetate solution (component B), starting with 8% A for 2 min, then changing A from 8 to 90% in 18 min, at last returning to 8% A in 1 min. The column temperature was maintained 30 °C, the flow rate was 1.0 ml/min and the detection wavelength was 254 nm.

### Biofilm assay

The ability to form biofilms was analyzed by a highly reproducible 96-well plate assay as described earlier [32]. Overnight cultures of *P. chlororaphis* HT66 and its mutants were diluted with 0.01 M phosphate-buffered saline (PBS) to 10^6^ CFU/ml, and 100 μl diluted cultures were seeded into a 24-well plate followed by incubation at 28 °C for 24 h under static conditions. After the designated time, the cultures were gently removed by pipetting and each well was washed twice with 150 μl sterile PBS. Afterwards, 200 μl of 1% (w/v) crystal violet (CV) was added to each well to stain bacterial biofilm, and the plate was incubated at 28 °C for 20 min. The CV was then rinsed with 500 μl 95% ethyl alcohol and the amount of biofilm was quantified by measuring the optical density at 595 nm using Microtitre plate reader (Victor™ X series multi-label plate reader).

### In-vitro antifungal activity

*Pythium ultimum* [33] was used to detect the antifungal activity of HT66 and its mutant derivatives. A mycelium disc of fungus was scrapped from the colonies grown on an agar plate, and spotted at the left of the agar plate, whereas the HT66 and its mutants were inoculated at the right of the agar plate. Fungal growth was monitored following incubation in darkness for 2 weeks at 28 °C. Three replicate plates were tested for each treatment.

## Results

### Detection and identification of AHLs produced by HT66

*Chromobacterium violaceum* CV026 and *A. tumefaciens* NTL4 plate assay were used to analyze the AHL-containing extracts of strain HT66. The CV026 indicator strain responds only to medium-chain alkanoyl-homoserine lactones (alkanoyl-HSLs) by producing purple spots, while NTL4 (pZLR4) responds strongly to 3-OH and 3-oxo-substituted HSLs, which react with X-gal resulting in blue pigment production. After the addition of AHL extracts to CV026 and NTL4 plates, the NTL4 assay showed blue pigment, while CV026 did not produce purple pigment, suggesting that the AHLs produced by HT66 might be 3-OH and 3-oxo-acyl-HSLs.

**Fig. 1:**
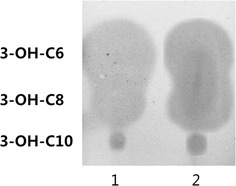
Detection of AHLs produced by *Pseudomonas chlororaphis* HT66 and *E. coli* with the plasmid pME-*phzI* using *Agrobacterium tumefaciens* NTL4. Lane 1, AHLs extract from HT66 and lane 2, AHLs from *E. coli* (pME-*phzI*)

**Fig. 2:**
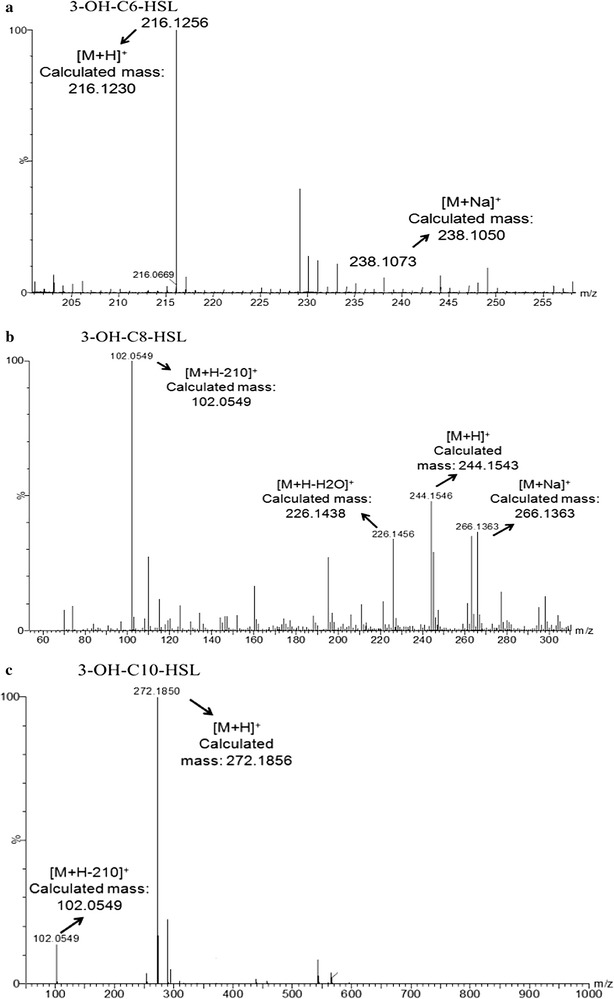
Identification of AHLs produced by *Pseudomonas chlororaphis* HT66 using LC–MS/MS analysis, **a** 3-OH-C6-HSL **b** 3-OH-C8-HSL and **c** 3-OH-C10-HSL

Further, ethyl acetate extracts from HT66 culture supernatants were separated by C18-reverse TLC and then overlaid with the biosensor NTL4 strain. Notably, three blue spots were detected from the extracts of HT66 (Fig. 1). The detected compounds were compared with reported known R_f_ values [34] and speculated that AHLs might be 3-oxo or 3-OH-acyl-HSLs with side chains ranging from 6 to 10 carbons. To identify the signals more precisely, extracts were separated by preparative TLC, and then the corresponding parts were scrapped and dissolved in the sample for HPLC–MS/MS analysis. It is known that every member of the AHL family gives a particular lactone ring at a nominal mass of *m/z* 102.05 [35], which results from the cleavage of homoserine lactone ring at the *N*-acyclic side chain. This characteristic ion was the key to analyze AHLs by HPLC–MS/MS. After MS/MS analysis, we found that three ions generated a peak for the characteristic ion at *m/z* 102.05 and some other peaks of fragmentations (Fig. 2a–c). For example, the 3-OH-C8-HSL has a molecular weight of 243, and the detected mass fragmentation [M + H]^+^ was 244.1546, almost same as the calculated mass 244.1543. Moreover, the ion [M + H–H_2_O]^+^ (226.1456) and [M + Na]^+^ (266.1363) were also observed and confirmed that the AHL was 3-OH-C8-HSL. Combined with the results of TLC and bio-reporter experiments, we concluded that the AHLs produced by HT66 were 3-OH-C6-HSL, 3-OH-C8-HSL, and 3-OH-C10-HSL.

### PhzI of strain HT66 directs the synthesis of AHLs

The whole-genome shotgun sequence of the HT66 has been deposited in National Center for Biotechnology Information (NCBI) databases under accession number ATBG00000000. In order to study which gene participated in quorum sensing and phenazine biosynthesis, *luxI* and *luxR* homologous genes were searched in the genome of HT66. According to the comparison results, one gene displayed extremely high identity (99%) to the AHL synthesis gene of PCL1391. This gene encodes 196 amino acids, of which only one amino acid is different from *phzI* of PCL1391. At the same time, an HdtS homolog was discovered, which has a similarity of 83% with the *HdtS* in *P. fluorescens* F113. The *hdtS* encodes 257 amino acids that direct the synthesis of C6-HSL, C10-HSL, and 3OH-C14:1-HSL in F113. Additionally, we also found another possible AHL synthesis gene in HT66 genome, *CsaI*, which had been reported to produce C4-, C5- and C6-HSL in *P. chlororaphis* 30-84 [36]. Although *CsaI* gene only contains 204 bp but shows high identity (over 93%) to the *csaI* (1416 bp) in strain 30-84. The CsaI–CsaR is a second quorum sensing system in 30-84, which is supposed to regulate the cell surface properties in response to the signals produced by *CsaI*.

**Fig. 3:**
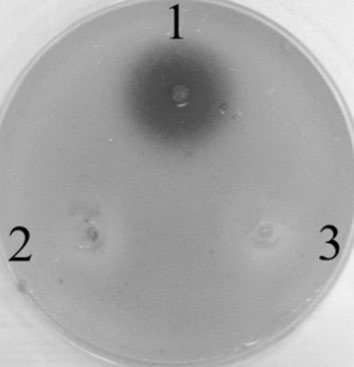
Detection of AHL extracts using *Agrobacterium tumefaciens* NTL4 plate bioassay. Samples contain extracts from cultures as follows (1) DH5*α* (pME-*phzI*) (2) DH5*α* (pME-*csaI*) and (3) DH5*α* (pME-*hdtS*)

To determine which genes produce the AHLs in HT66, the genes *phzI*, *casI* and *hdtS* were cloned and heterologous expressed in *E. coli* DH5α. A broad-host-range expression vector pME 6032 was used and the target gene was inserted between the *Eco*RI and *Xho*I sites, which was located in the downstream of the P_*tac*_ promoter. After induction with IPTG, the DH5α with plasmid pME-*phzI*, pME-*csaI* or pME-*hdtS* were cultivated overnight; the culture broths were then extracted with ethyl acetate and detected by *A. tumefaciens* NTL4 plate assay. Only the pME-*phzI* culture sample contained a detectable amount of AHLs (Fig. 3) and the AHLs produced by DH5α/pME-*phzI* was same as wild-type (Fig. 1). Whereas, the expression of *csaI* or *hdtS* in *E. coli* did not produce any signals judged by NTL4, which was totally different from the AHL synthase in strain 30-84 and F113 as discussed above.

To confirm our conclusion, we also constructed ∆*phzI*, ∆*csaI* and ∆*hdtS* mutants of HT66 through a non-scar gene knock-out method. Results showed that the *phzI*-deleted mutant completely loses the production of AHLs. On the other hand, as we assumed, the ∆*csaI* and ∆*hdtS* mutants exhibited no obvious difference with the wild-type and NTL4 assay showed blue pigment. Our results confirmed that *PhzI* was responsible for the synthesis of three AHLs (3-OH-C6, C8, C10-HSL) in HT66, which are irrelevant with CsaI or HdtS.

### *PhzI* and *PhzR* are essential for phenazine biosynthesis and over-expression of *phzI* improves the PCN production

**Fig. 4:**
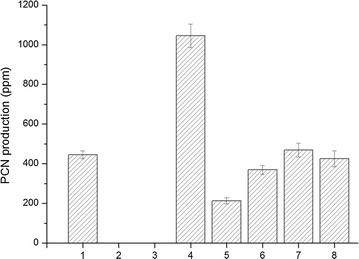
PCN production of HT66 and its mutants; (1) wild-type HT66, (2) HT66 ∆*phzI*, (3) HT66 ∆*phzR*, (4) HT66 (pME-*phzI*), (5) HT66 (pME6032), (6) HT66∆*phzI* (pME-*phzI*), (7) HT66 ∆*hdtS* and (8) HT66 ∆*csaI*

These two genes, *phzI,* and *phzR* are located just upstream of the phenazine biosynthesis gene cluster. In this work, we knocked out *phzI* from the HT66 genome to construct HT66∆*phzI* mutant through a non-scar deletion method. The results showed that deletion of *phzI* caused the complete loss of PCN biosynthesis as well as the AHLs synthesis (Fig. 4); whereas the constitutive expression of *phzI* restored the production of PCN and AHLs in ∆*phzI* mutant by transforming into the pME6032-*phzI* plasmid, which could produce up to 83% of the PCN concentration in wild-type. These facts suggested that PCN production of HT66 was regulated by *phzI* and is also responsible for the synthesis of AHLs. Similar to HT66∆*phzI* strain, the HT66∆*phzR* derivative was also unable to produce PCN, demonstrating that the phenazine biosynthetic cluster was regulated by PhzR receptor protein.

To investigate the effects of exogenous AHLs on PCN production in HT66, we transformed the *phzI* over-expressed plasmid into the wild-type strain, and the empty plasmid was also transformed as a control. Results evidenced that the HT66/pME-*phzI* had a higher level of PCN up to 1045 mg/l, which was almost 2.3- and 4.9-folds higher than wild-type and control strain (HT66 carrying the empty pME6032 plasmid). Thus, the results implied that exogenous AHLs production had improved the PCN production.

The absence of PCN production in ∆*phzI* or ∆*phzR* mutants indicated that a functional expressed quorum-sensing system was essential for the activation of the *phz* operon. The continuous over-expression of *phzI* resulted in an elevated level of PCN in wild-type HT66 strain. In addition, the PCN titers of ∆*csaI* and ∆*hdtS* mutants were detected to be 425 and 469 mg/l, respectively, and had statistically no difference in contrast with the wild-type HT66 strain.

### Effect of *phzI* and *phzR* on growth and biofilm formation

**Fig. 5:**
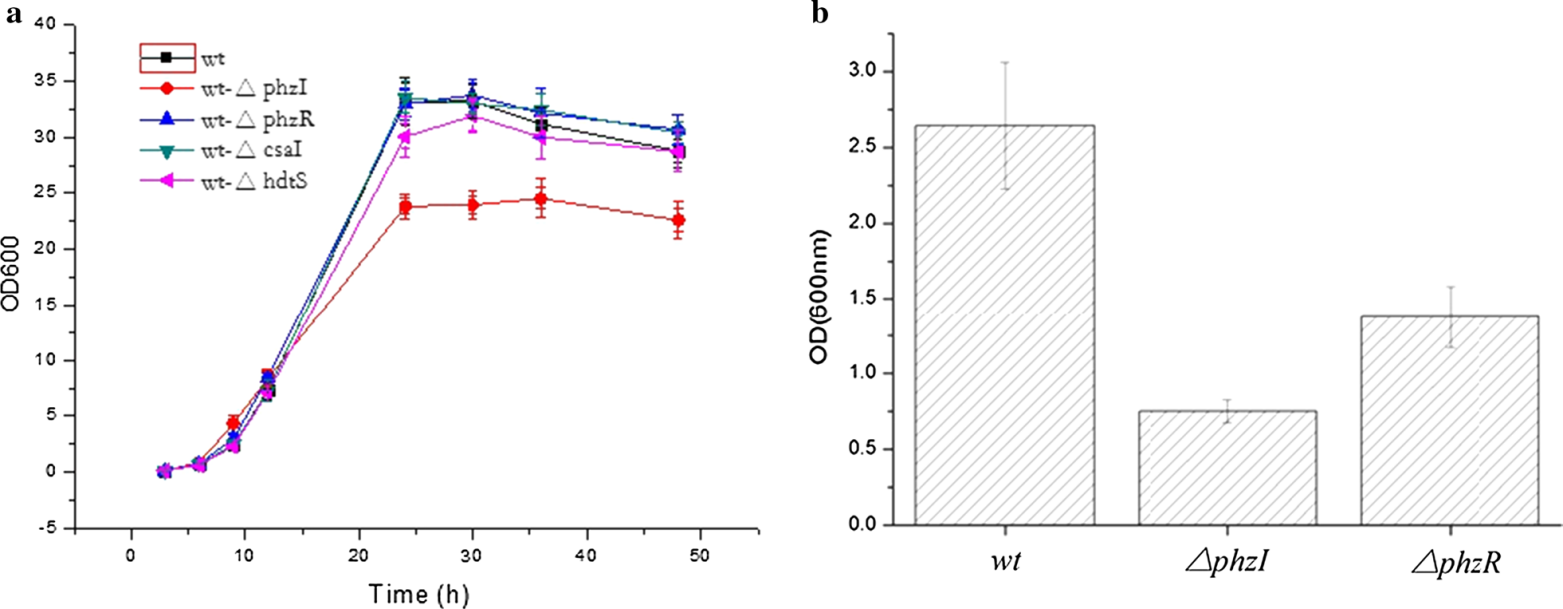
**a** The growth curves of HT66 and its derivatives and **b** biofilm formation in HT66 and its derivatives

A comparative growth profile results portrayed that the HT66 strain reached stationary phase at almost 24 h, and started to decline after 48 h (Fig. 5a). No noticeable difference was observed between the growth of wild-type strain and its ∆*phzR* mutant. While ∆*phzI* mutant had a lower cell density in stationary phase compared to wild-type, which might be correlated with the absence of signals and negative impact on communication between microorganisms.

Biofilm structures, which protect bacteria from various physical and chemical stresses, are the major reason for bacterial persistence during chronic infections [37]. Quorum sensing regulation of swarming and DNA release has been reported, which play important roles in *P. aeruginosa* biofilm development. On the other hand, QS signaling and transcription of genes are connected with the biofilm matrix biosynthesis [38]. Reports have shown that in some bacteria phenazines were not limited to secondary metabolites but also could function as cell signals [39]. We used 24-well plate assay to assess the biofilm formation ability of HT66 strains and its mutant derivatives. Compared with the wild-type, a significant decrease in biofilm production was observed in ∆*phzI* and ∆*phzR* mutants (Fig. 5b). The biofilm formation of ∆*phzI* was markedly damaged and recorded only 34% amount of the wild-type. It also appeared delicate and loose in a structure following stained with crystal violet. The findings indicated that PhzI–PhzR system plays a significant role in biofilm formation in strain HT66.

### Effect on colonial morphology and antifungal activity

On specialized colonial morphology medium (KB medium), the mutants had almost the identical growth rate as the wild-type to form colonies (Additional file 1: Figure S1). The colonies of HT66 were observed to be protruded, neat edge, and round with a semi-diameter of about 0.6 cm after 36 h growth. The surface was smooth with little viscosity. After inoculated on a plate for 48 h or a longer period, the wild-type colonies turned yellow and produced green pigment on the surface. The ∆*phzI* and ∆*phzR* mutants had no discernible changes in the morphology of colonies; however, their colonies appeared milky white and had no green pigment due to the lack of PCN production.

*Pythium ultimum* was chosen for antifungal activity experiment. HT66 and its mutants were inoculated on PDA agar plate, simultaneously with the pathogenic fungi on the other side. After cultivation together for 2 weeks, the development of pathogenic fungi was considerably inhibited by the wild-type presumably due to PCN production (Additional file 1: Figure S1). In contrary, the PCN-deficient mutant’s ∆*phzI* and ∆*phzR* could not inhibit the growth of mycelia. It is illustrated that the absence of *phzI*–*phzR* system reduced the antifungal activity of strain HT66 indicating the significance of PCN production in the biological control of soil-borne crop diseases.

## Discussion

In this study, thin-layer chromatography and liquid chromatography-mass/mass spectrometry techniques were applied to identify three AHLs (3-OH-C6, C8, C10-HSL) in *P. chlororaphis* HT66. Strain HT66 was a newly discovered phenazine-producing *P. chlororaphis* from rice rhizosphere, and the characteristic AHLs synthesis potential renders it distinguishing from other *Pseudomonads*. At first, we found three possible genes from the genome sequence that might produce AHLs. By heterologous expression in *E. coli* and non-scar deletion, *phzI* was evidenced to be the only responsible gene for the synthesis of these signals in HT66, while negating the contribution of *csaI* or *hdtS*. The knocking out of *csaI* or *hdtS* in HT66 has no obvious effect on PCN production and growth profile of HT66. In *P. aureofaciens* 30-84, CsaI–CsaR was not required for the expression of the gene involved in phenazine biosynthesis [36]. In *P*. *fluorescens* F113, *hdtS* was able to direct the synthesis of AHLs in strain F113. Nevertheless, the protein HdtS not belongs to either of the known AHL synthase families (LuxI or LuxM) and has a relationship with the lysophosphatidic acid acyltransferase family [40].

**Fig. 6:**
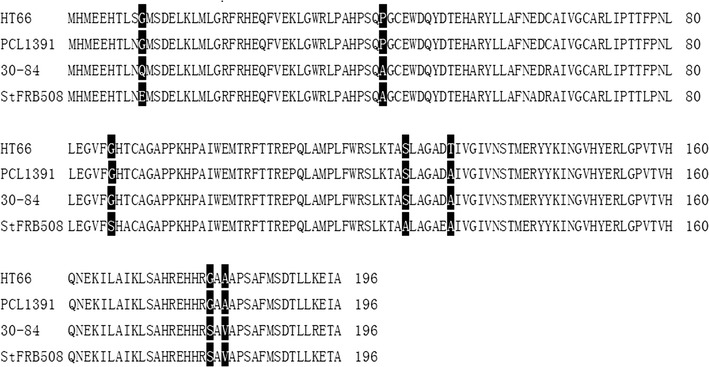
PhzI protein sequences of *Pseudomonas chlororaphis* HT66, PCL1391, *P. chlororaphis* 30-84 and *P. chlororaphis* subsp. *aurantiaca* StFRB508. The PhzI proteins were highly similar and closely related. Amino acids that are different in these four proteins are shown in black blocks

In Table 2, *phzI* in several *Pseudomonas* were compared with that in HT66, including their signals products. PhzI proteins of HT66 and PCL1391 were found to be similar as high as 99% (Fig. 6). It was interesting to note that C4 and C6-HSL were the products of the *phzI* gene in PCL1391, while HT66 produces 3-OH-C6-HSL, 3-OH-C8-HSL, and 3-OH-C10-HSL. The AHLs discrepancy was due to a different amino acid in PhzI proteins of HT66 and PCL1391 and noted to be threonine and alanine respectively, at position 125. Additionally, PhzI of strain 30-84 was also compared with that of HT66. The results show they shared almost 94% identical sequences (Fig. 6). Among the 197 amino acids sequence, there are five differences between PhzI HT66 and PhzI 30-84, and these five changes were: Glycine to Glutamine at position 10, Proline to Alanine at position 42, Threonine to Alanine at position 125, Glycine to Serine at position 179 and Alanine to Valine at position 181. As a consequence, AHLs produced by PhzI 30-84 were not only 3-OH-C6, C7, C8, C10-HSL but also C6-HSL and small amounts of C5- and C8-HSL. Regarding strain StFRB 508, there were 7 amino acids changes between PhzI 508 and PhzI HT66. PhzI 508 catalyzes the biosynthesis of C6-HSL as well as 3-OH-C6-HSL; whereas the second AHL synthase, AurI produces C4 and C6-HSL. In addition, we compared the *phzI* similarity of several phenazine-producing strains with HT66. The results revealed that these *phzI* genes were functionally same and were strongly conserved with the high sequence identity (Table 3).

**Table 2:** Primers used in this study

Primer name	Primer sequence (5′–3′)
*phzI*-F1	TCGGAATTCATGCACATGGAAGAGCACA
*phzI*-R1	TCGCTCGAGTCAAGCTATCTCTTTCAATAATGT
*csaI*-F1	TCGGAATTCATGGCGCGGAGCCGGCT
*csaI*-R1	TCGCTCGAGCTACTCCCTGAGCGCCTGA
*hdtS*-F1	TCGGAATTCATGTCGATCCTGCAGGCAATCAGAA
*hdtS*-R1	TCGCTCGAGTCAGATGGCCATTTTGTCCG
*ΔphzI*-F1	CCGGAATTCCGGACTGAAGGTTGCTGAGAG
*ΔphzI*-R1	TTACTATCTCCGAGTCGACCATCGAAGGCGACAGTTT
*ΔphzI*-F2	GGTCGACTCGGAGATAGTAAATGCCCCTC
*ΔphzI*-R2	CCCAAGCTTCGGTTTGATTTCTTTGCCTACGG
*ΔphzR*-F1	CCGGAATTCATGGAAGAGCACACACTGAG
*ΔphzR*-R1	TGTCACATTGAGGGTCTTGCATTTACTATCTCCGAGT
*ΔphzR*-F2	GCAAGACCCTCAATGTGACAGCCGTAAA
*ΔphzR*-R2	CCCAAGCTTTTGGCGAAGTTCAAGATGATCATT
*ΔcsaI*-F1	CCGGAATTCCAGTTGACCGAGGAAGGC
*ΔcsaI*-R1	TCTACTCCCTGAGCGCCTGAGTAGGTAAAGACACTTG
*ΔcsaI*-F2	TCAGGCGCTCAGGGAGTAGACCAGCG
*ΔcsaI*-R2	CCCAAGCTTCGATCCTGTCGTACCTGGC
*ΔhdtS*-F1	CCGGAATTCACGACTCCGACGCTTACATC
*ΔhdtS*-R1	CGGTACACAGGTTATCCACAAGGATGTCAGAAGAACT
*ΔhdtS*-F2	TGTGGATAACCTGTGTACCGTTTTAGCGGAAATCGC
*ΔhdtS*-R2	CCCAAGCTTCCTCGACGACGATGCC

**Table 3:** AHLs produced by different *Pseudomonas* strains

*Pseudomonas*	Similarity of *PhzI* compared with HT66 (%)	AHLs synthesized by *PhzI*	References
*Pseudomonas chlororaphis* HT66	N/A	3-OH-C6, C8, C10-HSL	This study
*Pseudomonas chlororaphis* PCL1391	99	C6-HSL	Gigard et al. [49]
*Pseudomonas chlororaphis* 30-84	95	C6-HSL, 3-OH-C6, C7, C8, C10-HSL	Khan et al. [50]
*Pseudomonas chlororaphis* subsp. *aurantiaca* StFRB508	94	C6-HSL, 3-OH-C6-HSL	Morohoshi et al. [41]
*Pseudomonas* sp. G5(2008b)	94	C4, C6, C8-HSL	Li et al. [51]
*Pseudomonas chlororaphis* GP72	94	C4, C6-HSL	Huang et al. [52]

Apart from the identification and production of AHLs in HT66, this study found that the over-expression of *phzI* had improved the level of PCN by 2.3-folds compared with the wild-type strain. More importantly, PCN production was abolished following the inactivation of *phzI* or *phzR* genes in HT66 indicating their roles in regulating the expression of the phenazine biosynthesis gene cluster. Previously, Morohoshi et al. [41] reported that the mutation in phzI caused a considerable reduction in phenazine biosynthesis *by P. chlororaphis* subsp. *aurantiaca*. On the other hand, no phenazine production was recorded in the triple mutant of phzI, aurI, and csaI (508ΔPACI). Noticeably, phenazine production was supposed to be strongly stimulated by PhzI-mediated AHLs than produced by AurI and CsaI that only induced a slight stimulation. Though, *Pseudomonas* sp. CMR12a and *P. chlororaphis* subsp. *aureofaciens* 30-84 display a second QS system apart from phzI/phzR system, phenazine biosynthesis was merely regulated by the AHLs produced by PhzI [36, 42, 43].

In order to appraise the involvement of AHL production to the biocontrol capacity of StFRB508, *P. ultimum* was used in this study. After cultivation together for 2 weeks, the development of pathogenic fungi was considerably inhibited by the wild-type presumably due to PCN production confirming the strain as an effective biocontrol agent. In *P. chlororaphis* PA23, it was examined that the biofilm development was reduced by approximately fivefold in the QS-deficient mutants and the motility was also altered [44]. Similarly, the biofilm formation in *phzI* and *phzR* deletion strains of HT66 had a decrease in different extents. With the absence of PCN, the antimicrobial ability was influenced when grew against the *P. ultimum* in vitro.

## Conclusions

In conclusion, we identified three kinds of AHLs (3-OH-C6-HSL, 3-OH-C8-HSL, 3-OH-C10-HSL) in *P. chlororaphis* HT66 by TLC and HPLC–MS/MS analyses. The production of AHLs exhibiting the activating role in phenazine biosynthesis gene cluster was only catalyzed by PhzI. In comparison to wild-type HT66, PCN production was 2.3-folds improved by over-expression of *phzI*, however, *phzI* or *phzR* mutant did not produce PCN. In addition, the growth, morphology and biofilm formation were all under the control of the PhzI–PhzR regulatory system. These results revealed that PhzI–PhzR system plays a pivotal role in PCN production and the biocontrol activity of HT66.

## Additional files

**Additional file 1: Figure S1.** **a** Colonial morphology changes in wild-type HT66, HT66∆*phzI* and HT66∆*phzR* strains during 7 days and **b** Influence of antifungal activity of HT66 and its mutants on the growth of *Pythium ultimum*. *P. ultimum* was spotted at the left of the PDA plate, whereas HT66 and its mutants were inoculated on the right side.
