# Supplementary material for: Identification, synthesis and regulatory function of the N-acylated homoserine lactone signals produced by Pseudomonas chlororaphis HT66
Source: Microb Cell Fact. 2018 Jan 22;17:9. doi: 10.1186/s12934-017-0854-y (PMC5776774; doi:10.1186/s12934-017-0854-y)
Supplement: Supplementary file 1 — Additional file 1: Figure S1. a Colonial morphology changes in wild-type HT66, HT66∆phzI and HT66∆phzR strains during 7 days and b Influence of antifungal activity of HT66 and its mutants on the growth of Pythium ultimum. P. ultimum was spotted at the left of the PDA plate, whereas HT66 and its mutants were inoculated on the right side. [file 12934_2017_854_MOESM1_ESM.docx]

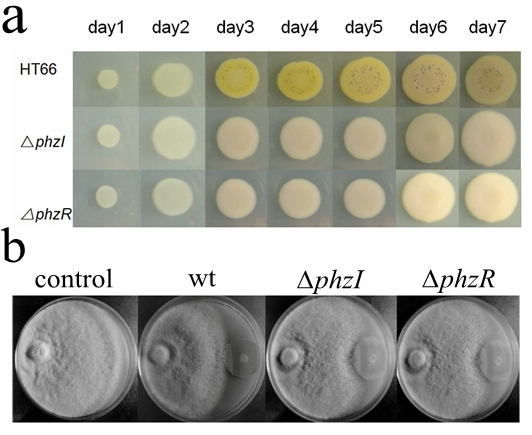


Figure S1A) Colonial [morphology](app:ds:morphology) changes in wild-type HT66, HT66∆*phzI* and HT66∆*phzR* strains during seven days and B) Influence of antifungal activity of HT66 and its mutants on the growth of *Pythium ultimum.* *P. ultimum* was spotted at the left of the PDA plate, whereas HT66 and its mutants were inoculated on the right side.
